# Programmatic implications of the TUMIKIA trial on community-wide treatment for soil-transmitted helminths: further health economic analyses needed before a change in policy

**DOI:** 10.1186/s13071-020-3977-7

**Published:** 2020-02-27

**Authors:** Hugo C. Turner, Donald A. P. Bundy

**Affiliations:** 1 Oxford University Clinical Research Unit, Wellcome Africa Asia Programme, Ho Chi Minh City, Vietnam; 2 Centre for Tropical Medicine and Global Health, Nuffield Department of Medicine, University of Oxford, Oxford, UK; 3 London School of Hygiene and Tropical Medicine, London, UK

**Keywords:** Soil-transmitted helminths, Policy, Deworming, Community-wide treatment, School-based treatment, Cost

## Abstract

School-based deworming programmes are currently the main approach used to control the soil-transmitted helminths (STHs). A key unanswered policy question is whether mass drug administration (MDA) should be targeted to the whole community instead, and several trials in this area have been conducted or are currently on-going. A recent well-conducted trial demonstrated that successful community-wide treatment is a feasible strategy for STH control and can be more effective than school-based treatment in reducing prevalence and intensity of hookworm infection. However, we would argue that it is vital that these findings are not taken out of context or over generalised, as the additional health benefits gained from switching to community-wide treatment will vary depending on the STH species and baseline endemicity. Moreover, community-wide treatment will typically be more expensive than school-based treatment. The epidemiological evidence for an additional benefit from a switch to community-wide treatment has yet to be proven to represent “good value for money” across different settings. Further work is needed before changes in policy are made regarding the use of community-wide treatment for STH control, including comprehensive assessments of its additional public health benefits and costs across a range of scenarios, accounting for the presence of alternative treatment delivery platforms.

## Letter to the Editor

School-based deworming programmes are currently the main approach used to control the soil-transmitted helminths (STHs) [1]. A key unanswered policy question is whether mass drug administration (MDA) should be targeted to the whole community instead, and several trials in this area have been conducted or are currently on-going [2–4]. For example, Pullan et al. [4] recently published an impressive and comprehensive trial which demonstrated that community-wide treatment of STHs in Kenya is possible and can be more effective for reducing the prevalence and intensity of hookworm (the main helminth in the communities studied). Specifically, they observed that after 24 months and two rounds of treatment, the overall prevalence of hookworm declined from 18.6% to 13.8% within the school-based group compared to 17.9% to 8.0% within the community-wide treatment group. Their findings also highlighted the equity of the community delivery approach and its ability to reach the poorest and most marginalised communities.

An important question arises whether these results support the policy contention that community-wide treatment is cost-effective, and hence should replace school-based treatment as the standard method of delivery for STH control.

In terms of programme effectiveness, there are two key outcomes of interest: additional reductions in morbidity and contribution to breaking transmission. Helminth morbidity is typically correlated to the intensity of infection, and individuals with moderate to heavy intensity infections experience the majority of STH-related morbidity [5–7]. Due to the non-linear relationship between STH prevalence and intensity [8], when the prevalence of infection falls below 20%, the prevalence of these moderate to heavy intensity infections is expected to be relatively low [9]. Due to this, it is likely that the observed additional reductions in prevalence achieved by using community-wide treatment would only lead to small additional gains in averted morbidity. These findings should not be overgeneralised, and the observed benefit may have been partly reduced due to the previous community-wide treatment for lymphatic filariasis that occurred before the study started [4]. In addition, mathematical models have also projected larger benefits of community-wide treatment in settings that have high baseline prevalence of hookworm [10]. However, the additional benefits of community-wide treatment would likely be even lower where *Ascaris lumbricoides* and *Trichuris trichiura* are more prevalent, since unlike hookworm, these species typically have much lower levels of infection in adults relative to children [7, 10–12]. Consequently, the additional health benefits gained from switching to community-wide treatment will vary depending on which STH species are endemic, and the baseline level of endemicity [10]. It is likely that in at least some settings both school-based and community-wide approaches can sustain infection below a level likely to be associated with significant morbidity.

A potential further benefit of community-wide treatment for STHs is that it may contribute to breaking transmission [13, 14]. Typically, treatment is followed by re-infection [15], but it is also theorized that sufficient treatment would result in the collapse of transmission (i.e. stochastic extinction), thus removing the need for further MDA. Modelling studies have estimated a breakpoint of around 2% prevalence for hookworm [16]. However, the reductions in prevalence under community-wide treatment in the TUMIKIA study did not approach this estimated breakpoint within the trial period and the studies available to date in this area have not definitively proven breaking transmission is possible with either school-based or community-wide treatment. The TUMIKIA study also indicates that achieving the projected high coverage and compliance levels estimated to be required to break transmission in some settings in a programmatically realistic time horizon could be difficult within many control programmes.

A major consideration regarding the use of community-wide treatment is its cost [17]. Even when the per treatment costs are lower relative to school-based strategies, the total cost will typically be higher because more individuals are treated. This is illustrated with an example in Table 1. Although this is a crude calculation, it indicates that even with conservative, at-scale estimates, the total cost of community-wide treatment is likely to be more expensive than the total cost of school-based treatment. Does the additional benefit outweigh this larger investment and greater complexity of implementation: is community-based treatment good value for money or cost-effective? Further studies are needed to obtain more accurate estimates of the difference in these costs [17]. There is potential for community-wide treatment to be cost-saving in the long term if it can stop the need for further treatment [10], but this is contingent on the interruption of transmission in an appropriate timescale, which has yet to be demonstrated.

**Table 1:** Hypothetical case study of the estimated financial costs of using different treatment strategies within the Kenyan national STH control programme

Strategy	Number treated	Assumed cost per treatment (US$)	Estimated total financial cost per year (US$)
School-based treatment	6 million children [23]	0.30^b^–0.56^c^	1.8–3.4 million
Community-wide treatment	14 million individuals^a^	0.32^d^–0.46^e^	4.4–6.4 million

^a^Approximated based on demographic data from the World Bank [24]

^b^Based on the WHO MDA cost benchmark model [25]

^c^Estimate from Evidence Action (a programmatic estimate for 2015) [23]

^d^Based on the estimate from the TUMIKIA trial [4]: routine scenario (excluding the research costs) relating to whole county (i.e. estimate at scale). US$0.025 per treatment was added for the cost of albendazole [4]

^e^Based on the estimate from the TUMIKIA trial [4]—routine scenario (excluding the research costs) relating to trial areas only. US$0.025 per treatment was added for the cost of albendazole [4]

To conclude, the TUMIKIA study is an important and well-conducted trial which demonstrates that successful community-wide treatment is a feasible strategy for STH control and can be more effective than school-based treatment in reducing prevalence and intensity of hookworm infection. However, these results should not be taken out of context, and achieving marginally greater reductions in already low prevalence settings did not necessarily lead to significant public health benefits or break transmission. Yet, community-wide treatment will typically be more expensive than school-based treatment. This does not mean that a community-wide treatment is never cost-effective [18, 19], but it does indicate that there are many contexts where switching to community-wide treatment would not yield notable public health benefits compared to remaining with school-based treatment. The policy implications of these results should not be over-interpreted or be extrapolated to all contexts. Ongoing studies (such as DeWorm3) are assessing the capacity of community-wide treatment for breaking STH transmission [3], which could importantly change the policy implications, but this approach currently remains unproven. Importantly, community-wide mass treatment is not the only strategy to provide treatment to adults and it is probable that building on established health system approaches (such as using child health days and antenatal clinics) to deliver treatment could offer a way of treating adults at a lower cost [20, 21]. Further work is needed before changes in policy are made regarding the use of community-wide treatment for STH control, including comprehensive assessments of its additional public health benefits and costs across a range of scenarios, accounting for the presence of alternative treatment delivery platforms for reaching at-risk groups other than school-aged children [21, 22].

## Data Availability

Not applicable.

## Abbreviations

STH: soil-transmitted helminth
MDA: mass drug administration
